# Preferences for long‐acting pre‐exposure prophylaxis among gay, bisexual and other men who have sex with men in Taiwan: findings from the 2021 HEART Survey

**DOI:** 10.1002/jia2.26163

**Published:** 2023-09-07

**Authors:** Jing‐Hao Hsu, Stephane Wen‐Wei Ku, Tsai‐Wei Chen, Chia‐Wen Li, Poyao Huang, Huei‐Jiuan Wu, Adam Bourne, Carol Strong

**Affiliations:** ^1^ Department of Public Health College of Medicine National Cheng Kung University Tainan Taiwan; ^2^ Division of Infectious Diseases Department of Medicine Taipei City Hospital Renai Branch Taipei Taiwan; ^3^ HIV Education and Research Taiwan (HEART) Association Taipei Taiwan; ^4^ Infection Control Center and Department of Internal Medicine National Cheng Kung University Hospital College of Medicine National Cheng Kung University Tainan Taiwan; ^5^ Institute of Health Behaviors and Community Sciences National Taiwan University Taipei Taiwan; ^6^ The Kirby Institute UNSW Sydney Sydney New South Wales Australia; ^7^ Australian Research Centre in Sex Health and Society La Trobe University Melbourne Victoria Australia

**Keywords:** Asia, gay, bisexual and other men who have sex with men (GBMSM), HIV prevention, policy, pre‐exposure prophylaxis (PrEP), retention

## Abstract

**Introduction:**

While various antiretrovirals have been studied as potential candidates for long‐acting pre‐exposure prophylaxis (PrEP), the bimonthly injectable cabotegravir*—*the first long‐acting form of PrEP—was approved in 2021. Event‐driven (ED) PrEP has been the most prevalent dosing regimen among gay, bisexual and other men who have sex with men (GBMSM) in Taiwan, providing a unique setting to observe the preferences for long‐acting PrEP in a community where the daily regimen is not the mainstream method. This study aimed to determine the preferences for the different forms and dosing intervals of long‐acting PrEP that are currently in the development pipeline.

**Methods:**

We conducted a survey in 2021 by convenience sampling the users of social networking applications for GBMSM in Taiwan. Our survey included questions on sexual behaviours, current PrEP regimens and the preferences for potential candidates of long‐acting PrEP, such as implants, intramuscular and subcutaneous injections. We compared the Likert‐scale preference ratings for potential long‐acting options, and conducted logistic regression analysis to examine the factors associated with a preference for bimonthly intramuscular injections (2M IM) over ED and daily PrEP regimens, respectively.

**Results:**

A total of 1728 responses were eligible for analysis. Three percent of respondents (*n* = 52) were daily PrEP users; 11.5% (*n* = 198) were ED PrEP users. When not considering cost, current PrEP users—regardless of their original dosing regimen—were most likely to express preferences for monthly oral PrEP, followed by a 6‐month subcutaneous injectable (6M SC) and 2M IM. However, among non‐current PrEP users, monthly oral PrEP was the most preferred form, followed by ED, daily oral and 6M SC injectable. Multivariable logistic regression revealed that current daily users, those willing to take PrEP in the next 6 months and those with more sex partners in the last 12 months had a significant correlation with preferences for the 2M IM injectable over the ED PrEP.

**Conclusions:**

The monthly oral form was the most preferable long‐acting PrEP among GBMSM in Taiwan. Current daily PrEP users preferred the 2M IM injectable over the ED PrEP, which made the 2M IM injectable a potential alternative. Further studies should focus on how the cost and delivery affect PrEP preferences and their actual uptake.

## 1 INTRODUCTION

The next generation of pre‐exposure prophylaxis (PrEP) includes a variety of delivery mechanisms, such as injectable agents, rings or implants [1]. Of these, injectable PrEP is at the most advanced stage of development, particularly given that antiretroviral drugs can be made into long‐acting suspensions. This allows for the next generation of PrEP to be administered by intramuscular injection [2, 3, 4, 5, 6]. The U.S. Food and Drug Administration approved bimonthly injectable cabotegravir in December 2021 as the first long‐acting PrEP (long‐acting cabotegravir [CAB‐LA]) [7]. Other formulations and delivery sites are being tested for clinical efficacy, including monthly intramuscular injections, 6‐month subcutaneous injections and 12‐month subdermal implants [8, 9, 10].

PrEP was first introduced to Taiwan in 2016 and was followed by the official PrEP guidelines in 2019 [11]. In Taiwan, the majority of PrEP users are gay, bisexual or other men who have sex with men (GBMSM); among them, almost 70% use PrEP on an event‐driven (ED) dosing regimen [12]. This is due to the inclusion and early adoption of the ED regimen in the PrEP guidelines [11], which were based on factors, such as limited resources, price of the drugs and the preferences of the target population [12]. Self‐reported PrEP adherence was higher among daily regimen users compared to ED, but those who switched from daily to ED have the highest concerns regarding lower adherence [13]. Since lower adherence puts individuals at a higher risk of HIV infection, CAB‐LA may be considered an alternative option for PrEP users who previously used to take daily PrEP or ED.

Several studies have examined the preferences for PrEP administration among GBMSM in different countries [14]. When participants were asked to choose daily oral or long‐acting PrEP, their preferences for using long‐acting PrEP ranged from 25% to 34% in GBMSM in the United States, but ranged from 30% to 44% across countries in Africa, Asia and Australia [15, 16, 17, 18, 19]. Such differences may be influenced by factors, such as health insurance accessibility and cost, the original dosing regimen of oral PrEP or the specific social and cultural forces shaping the lives of GBMSM in each country [14]. Taiwan provides an important study context given the current high preference for ED PrEP [12]. Factors associated with using ED PrEP, such as lower frequency of condomless anal sex, may also be concerns for people to adopt long‐acting PrEP [20]. The preference for such innovation in a context that favours ED PrEP can shed light on the decision‐making process for other countries that are also in the process of upscaling PrEP uptake.

The present short report examined the preferences for different forms and dosing intervals of long‐acting PrEP currently in the development pipeline in a community sample of GBMSM. Such a community sample provided an opportunity to examine the preferences for long‐acting PrEP not only among current users, but also among those who had never used PrEP, and among those who have discontinued.

## 2 METHODS

An anonymous, cross‐sectional, online survey was conducted through advertisements on social networking applications commonly used by GBMSM in Taiwan. Participants were recruited if they met the following criteria: (1) male aged 20 years or above, and (2) lived in Taiwan. The following were not included in the analysis: those who lived with HIV, those whose IP addresses were duplicated and those who had not completed the survey responses. All data were collected between November and December 2021. This study was approved by the Institutional Review Board of the lead author (B‐ER‐107‐098). No incentives were provided to the participants for completing the survey.

### 2.1 Variables

Our survey included questions relating to the following: socio‐demographic characteristics, sexual behaviours, engagement in chemsex (i.e. using ecstasy, ketamine, methamphetamine, gamma hydroxybutyrate (GHB)/gamma butyrolactone (GBL) or mephedrone before or during sex in the past 3 months), HIV and sexually transmitted infection (STI) status, PrEP awareness and uptake, loneliness and sexual happiness.

With respect to their preferences for various PrEP formulations, participants were asked, “There are many long‐acting formulations of PrEP in the pipeline. Without considering the cost, how much do you prefer each long‐acting formulation listed below?” Response options included the following: one tablet taken orally every day (daily oral); two tablets taken orally before sex, then one tablet at 24 and 48 hours after sex (ED oral); one tablet taken orally every month (monthly oral); intramuscular injection given every month (monthly IM); intramuscular injection given every 2 months (2M IM); subcutaneous injection given every 6 months (6M SC); and small subcutaneous implant inserted every year (12M implant). Participants were asked to indicate their preference for each option on a scale from 1 (least preferred) to 10 (most preferred), and details of each option were provided considering their lack of awareness regarding some new PrEP regimens. Additionally, we asked for participants’ agreement on the price range of how much they were willing to pay for long‐acting PrEP: from $2000 to $28,000 New Taiwan Dollars (NTD) (1NTD ≅ 0.033 United States Dollars; in 2021, the median monthly income in Taiwan was around 42,000 NTD).

### 2.2 Statistical analysis

All respondents were listed into three different groups according to their current PrEP use: the daily use group, the ED group and the not currently using group. We depicted the differences in preferences for each regimen in a bar chart with the *p*‐value of a post hoc test. Factors associated with the preference of 2M IM over ED PrEP (Model 1) and the other model on 2M IM over daily PrEP (Model 2) were identified by univariable and multivariable logistic regression. Option 2M IM was used in the analysis because it was the first approved long‐acting option globally and possibly available in Taiwan. In our analysis, people who had equal ratings for 2M IM and the oral option were considered as not favouring 2M IM. The multivariable model included only those variables that were significant in the univariable analysis (*p* < 0.05). All data were analysed by R software version 4.1.2.

## 3 RESULTS

A total of 3066 responses were recorded in the online survey, of which 1728 respondents (56.4%) were eligible for analysis. The exclusion cases totalled 15 due to IP duplication, 1127 did not complete the survey, 44 were aged below 20 and 152 were living with HIV. Table 1 presents the socio‐demographic characteristics. Regarding current PrEP use, 3% took PrEP daily, 11.5% took ED PrEP and 85.5% were not using PrEP, which included 6.5% who had ever used PrEP previously and 79.1% who had never used PrEP.

**Table 1:** The sample profile of socio‐demographic characteristics, sexual behaviours, status of HIV and STIs, PrEP awareness and mental health (*N* = 1728)

	*n*	%		*n*	%
**Socio‐demographics**			**HIV awareness and STIs**		
Age (years old)			Awareness of own HIV serostatus		
20–30	876	50.7	negative	1500	86.8
> 30	852	49.3	unknown	228	13.2
Monthly income (NTD)			STIs in the past 3 months		
≤45,000	1192	69.0	Yes	162	9.4
>45,000	536	31.0	No	1566	90.6
Residence			**PrEP awareness**		
Living in one of the six major cities	1360	78.7	Pre‐survey PrEP awareness		
Others	368	21.3	Yes	1402	81.1
**Sexual behaviours**			No	326	18.9
Sexual partners in the past 12 months			PrEP willingness in the next 6 months		
0–5	1164	67.4	Yes	849	49.1
> 5	564	32.6	No	879	50.9
CLAI in the past 3 months			**Mental health**		
Yes	1040	60.2	Loneliness in the past 6 months		
No	688	39.8	Always	322	18.6
Chemsex in the past 3 months			Not always	1406	81.4
Yes	81	4.7	Sexual wellbeing in general		
No	1647	95.3	Happy	820	47.5
PEP in the past 12 months			Unhappy	908	52.5
Yes	222	12.8			
No	1506	87.2			

*Note: N* = 1728.

**Abbreviations**: ED, event‐driven; GBMSM, gay, bisexual and other men‐who‐have‐sex‐with‐men; HIV, human immunodeficiency virus; NTD, New Taiwan Dollars (1 NTD ≅ 0.033 United States dollar [USD]); PrEP, pre‐exposure prophylaxis; STIs, sexually transmitted infections.

**Figure 1:**
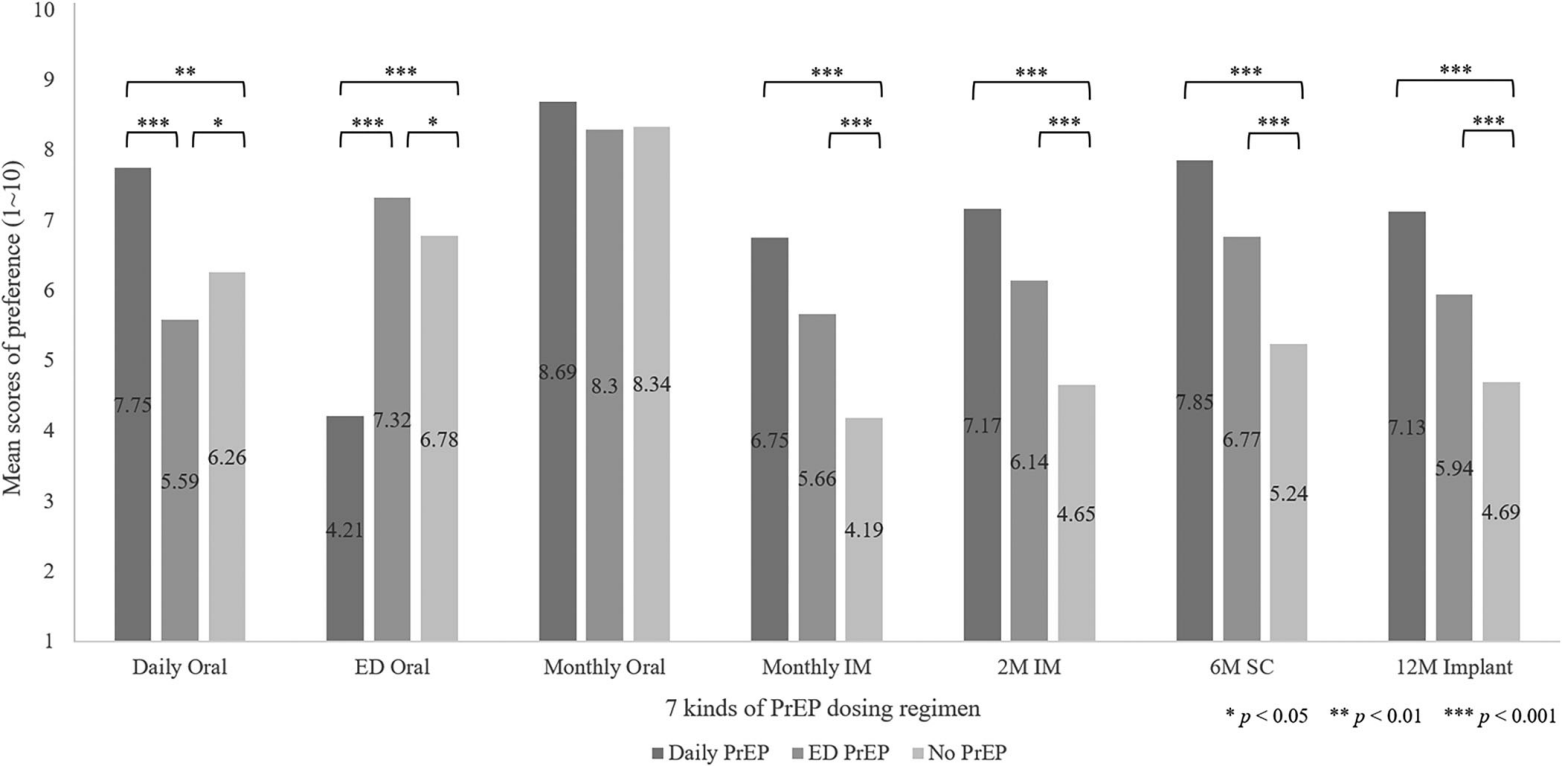
The mean of rating preference for daily, event‐driven or other potential long‐acting PrEP dosing regimens regardless of the cost. The X‐axis and the Y‐axis, respectively, showed seven kinds of PrEP dosing regimen and the mean score of preference as derived from a 10‐point Likert scale. The dark grey bar (the left side), grey bar (the middle) and light grey bar (the right side) represent the respondents of current daily group, current ED group and current PrEP non‐use group, respectively. Comparisons between the two groups were further conducted by post hoc Tukey honestly significant difference (HSD) test. Abbreviations: ED, event‐driven; IM, intramuscular injectable; M, month; SC, subcutaneous injectable. ^*^ *p* < 0.05; ^**^ *p* < 0.01; ^***^ *p* < 0.001.

Figure 1 displays the mean of each rating preference for daily, ED or other potential long‐acting PrEP dosing regimens. Current daily PrEP users preferred the monthly oral form the most, followed by 6M SC injectable, daily oral and 2M IM injectable. Current ED PrEP users preferred the monthly oral form the most, followed by ED, 6M SC injectable and 2M IM injectable. Respondents not currently using PrEP reported their preference for the monthly oral form the most, followed by ED, daily oral and 6M SC injectable.

Twenty‐three percent of respondents (*n* = 404) preferred 2M IM over ED PrEP. In Model 1 (Table 2), current daily PrEP users (adjusted odds ratio [aOR] = 2.26 vs. current ED users, 95% CI = 1.21–4.26), never use PrEP (aOR = 0.67 vs. current ED users, 95% CI = 0.47–0.96), PrEP willingness (aOR = 1.37, 95% CI = 1.06–1.76) and having a higher number of sex partners (aOR = 1.34, 95% CI = 1.05–1.72) was significantly associated with the preference of 2M IM injectable over ED PrEP. In Model 2, compared to current ED users, current daily users were 59% less likely to choose 2M IM over daily users when presented with both choices (95% CI = 0.21–0.78).

**Table 2:** Multivariable logistic regression for factors correlated with preference (*N* = 1728)

	Model 1 Preferred 2M IM over ED PrEP (*n* = 404, 23.4%)				Model 2 Preferred 2M IM over daily PrEP (*n* = 483, 28.0%)			
	Univariable		Multivariable^a^		Univariable		Multivariable^a^	
	OR (95% CI)	*p*‐value	aOR (95% CI)	*p*‐value	OR (95% CI)	*p*‐value	aOR (95% CI)	*p*‐value
**Socio‐demographics**								
Age (years old): >30 versus 20–30	1.11 (0.89−1.38)	0.370			1.02 (0.83−1.26)	0.842		
Monthly income (NTD): >45,000 versus <45,000	1.06 (0.83−1.34)	0.650			1.10 (0.88−1.38)	0.405		
Residence: living in one of the six major cities versus others	0.95 (0.72−1.24)	0.680			1.01 (0.79−1.32)	0.091		
**Sexual behaviours**								
Sexual partners in the past 12 months: >5 versus 0–5	1.67 (1.32−2.10)	<0.001^***^	1.34 (1.05−1.72)	0.020^*^	1.42 (1.34−1.77)	<0.002^**^	1.21 (0.95−1.52)	0.119
CLAI in the past 3 months	1.28 (1.01−1.61)	0.038^*^	1.02 (0.80−1.30)	0.873	1.26 (1.02−1.57)	0.035^*^	1.07 (0.85−1.35)	0.542
Chemsex in the past 3 months	1.32 (0.79−2.14)	0.280			1.02 (0.61−1.66)	0.927		
PEP in the past 12 months	1.19 (0.85−1.63)	0.301			1.25 (0.92−1.69)	0.152		
**HIV awareness and STIs**								
Awareness of own HIV serostatus: negative versus unknown	1.28 (0.91−1.83)	0.160			1.40 (1.02−1.97)	0.045^*^	1.17 (0.83−1.66)	0.373
STIs in past the 3 months	1.58 (1.10−2.23)	0.011^*^	1.28 (0.88−1.84)	0.187	1.06 (0.74−1.50)	0.752		
**PrEP awareness and uptake**								
Pre‐survey PrEP awareness	1.68 (1.23−2.32)	0.001^**^	1.34 (0.97−1.87)	0.079	1.53 (1.15−2.05)	0.004^**^	1.23 (0.91−1.67)	0.182
PrEP willingness in the next 6 months	1.77 (1.41−2.22)	<0.001^***^	1.37 (1.06−1.76)	0.014^*^	1.84 (1.49−2.86)	<0.001^***^	1.51 (1.20−1.91)	<0.001^***^
Current PrEP use								
daily versus ED	2.47 (1.32−4.63)	0.004^**^	2.26 (1.21−4.26)	0.011^*^	0.44 (0.22−0.84)	0.01^*^	0.41 (0.21−0.78)	0.009^**^
previous use versus ED	0.68 (0.40−1.12)	0.134	0.75 (0.44−1.26)	0.285	0.43 (0.26−0.70)	<0.001^***^	0.49 (0.29−0.80)	0.005^**^
never use versus ED	0.50 (0.37−0.70)	<0.001^***^	0.67 (0.47−0.96)	0.026^*^	0.36 (0.27−0.49)	<0.001^***^	0.49 (0.35−0.68)	<0.001^***^
**Mental health**								
Loneliness in the past 6 months: not always versus always	0.87 (0.66−1.16)	0.327			0.94 (0.73−1.24)	0.680		
Sexual wellbeing in general: unhappy versus happy	1.13 (0.91−1.42)	0.270			1.05 (0.85−1.29)	0.652		

**Abbreviations**: aOR, adjusted odds ratio; CI, confidence interval; CLAI, condomless anal intercourse; ED, event‐driven; IM, intramuscular injection; M, month; NTD, New Taiwan Dollar (1 NTD ≅ 0.033 United States dollar [USD]); OR, odds ratio; PEP, post‐exposure prophylaxis; PrEP, pre‐exposure prophylaxis; STIs, sexually transmitted infections.

^a^ Multivariable model only includes variables that were significant in the univariable (*p* < 0.05).

^*^ *p* < 0.05; ^**^ *p* < 0.01; ^***^ *p* < 0.001.

Regarding affordability, 67.9% of respondents were willing to pay less than 4000 NTD per injection in order to receive 2M IM injectable PrEP; 15.3% were willing to pay 4000–12,000 NTD per injection; 1.3% were willing to pay over 12,000 NTD per injection; 15.5% had no interest in paying for injectable PrEP.

## 4 DISCUSSION

The present study simultaneously compared preferences for seven different PrEP dosing regimens in Taiwan—a country where ED PrEP was highly popular among key populations. In terms of long‐acting formulations of PrEP, current PrEP users in Taiwan—regardless of their original dosing regimen—were most likely to express preferences for monthly oral PrEP, followed by 6M SC and 2M IM. This result of a preference for the oral PrEP regimen more than the injection regimen differs from research studies that have often reported higher preference for non‐oral PrEP regimen options—even though those studies had not included the monthly oral PrEP option for participants [15, 21–23].

The reasons why the oral PrEP regimens were much preferred in our sample were likely multifaceted and should be the focus of in‐depth qualitative enquiry. However, concern about injectable forms of medication to address other diseases and symptoms has been reported in Taiwan. For example, type 2 diabetic patients in Taiwan were often reluctant to use insulin injections, leading doctors to more commonly prescribe oral drugs [24]. Other possible reasons for a lower preference for injecting PrEP included having more concerns about the level of protection and the drug half‐life of long‐acting PrEP [19], or concerns for the duration of effectiveness [22]. In clinical trials, however, injectable PrEP has been proven to have a superior efficacy to oral PrEP regimens [4, 6]. Enhanced communication between healthcare providers and PrEP users is needed to convey accurate evidence about the effectiveness of long‐acting injectable PrEP.

There were still subgroups that preferred 2M IM over ED PrEP in our sample, including those with a higher willingness to use PrEP in general, and those with a higher number of sexual partners. Even though current daily users would choose daily oral over 2M IM, when provided with the choice of 2M IM and ED, they were willing to choose 2M IM over ED. Current daily users can thus be potential early adopters of injections. One reason may be that daily PrEP users in Taiwan were shown to have more condomless anal sex [20]. Our findings seem to indicate that the long‐acting IM PrEP may be an attractive alternative for those with a higher frequency of PrEP use. Compared to ED PrEP users who may not have a high demand for using PrEP, daily PrEP users and individuals with multiple sexual partners at higher risk of infection may be more viable candidates for PrEP and the protection that the injectable forms offer.

Our study also provides evidence regarding non‐current PrEP users’ preferences for long‐acting PrEP. This is a unique feature since the current literature has examined preferences mostly among PrEP users [15, 16, 17, 18, 19]. Non‐current PrEP users in our sample had even lower interest in injection options. The potential barriers retarding the use of innovative PrEP options, such as the perceived risk and willingness to start or resume PrEP—particularly the injection options—should be addressed in PrEP implementation policies.

With respect to future PrEP policy development in Taiwan, long‐acting injectables could be prioritized through public funding programmes for daily PrEP users or high‐risk individuals (i.e. people with multiple sexual partners, those with STIs or those who practice condomless anal sex). Although studies have shown that long‐acting injectables can improve adherence [25], overcoming rejection and increasing acceptance (such as using shared decision‐making) of long‐acting PrEP among most Taiwanese may be a challenge.

In terms of limitations, the ways in which the cost of long‐acting PrEP may shape preferences were not accounted for and should be the focus of further study. Future studies should also extend to other HIV‐affected groups in Taiwan, such as cisgender women, people who use drugs or transgender individuals. Another limitation is our use of a Likert scale to rate several long‐acting options for less cognitive burden and response times for online surveys. Other validated ways to rank preferences, such as best‐worst scaling or discrete choice experiments, are available and should be considered for future study [26].

## 5 CONCLUSIONS

The preference for injectable forms of PrEP among GBMSM in Taiwan is not as high as observed in studies in other countries. The monthly oral form was the most preferred long‐acting option, regardless of current PrEP use status. 2M IM PrEP could be an alternative to daily oral PrEP, especially for high‐risk individuals. The focus of further studies should include how the cost of PrEP regimens and the mode of delivery could affect users’ preferences and their actual uptake.

## COMPETING INTERESTS

SW‐WK receives support as a speaker and as a member of advisory boards for ViiV Healthcare, Gilead, MSD and Janssen. C‐WL receives support as a speaker and as a member of advisory boards for ViiV Healthcare, Gilead, MSD and Janssen. All other authors declare no competing interests.

## AUTHORS’ CONTRIBUTIONS

SW‐WK conceived the presented research idea and verified the underlying data. SW‐WK and T‐WC conducted the data collection, laboratory activities, and reviewed the collected data for quality and reliability. T‐WC analysed the data. SW‐WK, J‐HH and CS contributed to interpreting the results and took the lead in writing the manuscript. SW‐WK and CS were in charge of overall direction and planning. All authors provided critical feedback, shaped the research, analysed the manuscript and approved the final submitted manuscript.

## FUNDING

The study was funded by grants from the National Cheng Kung University Hospital, Tainan, Taiwan (NCKUH‐10902066), from the National Science and Technology Council (MOST NSTC 108–2636–B–006–004, NSCT 109–2636–B–006–004, NSTC 110–2636–B–006–011, NSTC 111–2636–B–006–011, NSTC 112–2636–B–006–007, NSTC 112–2314–B–006–081–MY3).

## Data Availability

The data that support the findings of this study are available from the corresponding author upon reasonable request.
